# Construction of a model for adolescent physical and mental health promotion based on the multiple mediating effects of general self-efficacy and sleep duration

**DOI:** 10.1186/s12889-023-17197-z

**Published:** 2023-11-20

**Authors:** Ru-bao Dong, Kai-yun Dou, Jie Luo

**Affiliations:** 1 School of Physical Education, Guizhou Normal University, Guizhou, China; 2 School of Psychology, Guizhou Normal University, Guizhou, China

**Keywords:** Health promotion, Adolescents, Physical activity, Mental health, Sleep duration, General self-efficacy

## Abstract

We developed a model for promoting adolescent physical and mental health (MH) to investigate the integrated pathways of physical activity (PA), MH, general self-efficacy (GSE), and sleep duration (SD) promotion among adolescents in China. The research hypotheses were tested using questionnaires, descriptive statistics, and validated factor analysis. The mean age of the respondents was 14.94 ± 1.76 years, the PA level was 2.49 [95% confidence interval (CI): 2.46–2.53], the GSE score was 2.60 (95% CI: 2.57–2.64), the SD was 436.52 min/day (95% CI: 432.89–440.15), and the total mean MH score was 1.72 (95% CI: 1.69–1.76) (model fitness index: χ^2^/df = 1.295, comparative fit index = 0.999, Tucker–Lewis index = 0.997, root mean square error of approximation = 0.014, standardized root mean square residual = 0.007). The SD accounted for 46.85% of the mediating effect. The adolescents exhibited moderately low PA levels, severe SD deficits, and high rates of overall MH abnormalities. Consequently, the constructed model for promoting adolescent physical health and MH was confirmed to be reliable; GSE and SD are significant factors mediating MH promotion.

More than 80% of the adolescent population is insufficiently physically active globally. Physical inactivity is a leading risk factor for noncommunicable diseases and death worldwide, increasing the risk of cancer, heart disease, stroke, and diabetes by 20–30%. It burdens society through the hidden and growing cost of medical care and loss of productivity [1]. Globally, an estimated 1 in 7 (14%) 10–19-year-olds and approximately 20% of children and adolescents have MH conditions, with suicide being the second leading cause of death among 15–29-year-olds. Approximately one in five people in post-conflict settings has an MH condition. These conditions can significantly impact all areas of life, such as school or work performance, relationships with family and friends, and the ability to participate in the community. The consequences of failing to address adolescent MH conditions extend into adulthood, impairing both physical health and MH and limiting opportunities to lead fulfilling lives as adults [2]. A study conducted in 24 countries and regions showed that 32–86% of adolescents achieved the recommended number of hours of sleep during school days, whereas 79–92% achieved them during non-school days. In the same study, in 15 countries, the average sleep duration (SD) of male students during school days exceeded that of female students [3]. In China, 90.8% of middle school students sleep < 9 h/day and 84.1% sleep < 8 h/day [4]. The decline in self-reported SD among American adolescents is concerning [5]. Approximately 70% of teens report getting less sleep [6], and the number of short-time sleepers continues to increase [7]. Asian adolescents tend to sleep later than North American and European ones, with a shorter total SD [8]. The lack of sleep, physical activity (PA), and serious MH problems among adolescents have become an intractable global problem. Together, they constitute a stumbling block to the sustainable development of society.

At the same time, PA has significant health benefits for hearts, bodies, and minds. Regular PA is proven to help prevent and manage noncommunicable diseases. It can also improve MH, quality of life, and well-being. Short SD (< 8 h at ages 13–18 years) has been linked to negative health outcomes, such as obesity, diabetes, hypertension, and depression among adolescents [9]. The World Health Organization’s definition of health includes the multidimensional concepts of physical health, MH, social adaptation, and moral health. Therefore, PA, MH promotion, and sleep deprivation interventions should be treated as integral components of health promotion. Comprehensive interventions should be implemented to achieve double the effect with half the effort. However, few studies have treated PA, SD, and MH as a whole and implemented comprehensive interventions.

Adolescence is a unique stage of human development and an important time for laying the foundations of good health. Adolescents need to acquire diverse knowledge and skills, leaving them with relatively little spare time. To more effectively implement time-saving and effective integrated comprehensive interventions for adolescent PA, MH, and sleep time deprivation, we used PA level, general self-efficacy (GSE), SD, and MH status to construct a mediating model of PA promoting MH, aiming to reveal different pathways by which PA promotes MH and provide new ideas and a theoretical basis for the integrated promotion of adolescent health.

## Theoretical foundation and model construction

### Theoretical basis

#### The direct contribution of PA to SD, GSE, and MH

PA is widely recognized as a key factor in preventing and managing mental illness, including conditions such as depression and anxiety, while also promoting MH and well-being [10]. Poor MH is negatively associated with PA levels [11]. PA exerts a protective and therapeutic influence on specific mental disorders, such as depression [12]; reduces anxiety symptoms, psychological stress, depression, self-esteem issues, and dependence; improves sleep quality and happiness; and alleviates fatigue and interpersonal and emotional challenges [13]. Interventions based on PA levels slightly lower than the recommended guidelines can yield significant MH benefits [14]. Moreover, there is evidence of a mutually reinforcing relationship among PA, SD, GSE, and MH. For example, participation in sports activities can reduce tension, anger, and depression among overweight adolescents, effectively promoting their overall well-being and self-satisfaction [15].

Engaging in physical exercise can enhance GSE, and this sense of GSE plays a mediating role between physical exercise and the MH of middle school students [16]. Physical inactivity increases the risk of inadequate SD [17]. These studies demonstrate that PA can promote GSE, SD, and MH. They also shed light on the amount of PA required to positively impact MH in adolescents; for instance, meeting the PA guidelines significantly correlates with overall positive psychology among adolescents [18].

#### Promotional effects of SD and GSE on MH

Individuals with low GSE are more prone to report MH issues compared to those with high GSE [19]. PA and sleep may be interdependent and impact health through interconnected pathways [18]. Inadequate SD is negatively associated with perceived MH [20]. It is common among adolescents and is linked to poor school performance and MH challenges, including mood swings, anxiety, and thoughts of suicide [21]. Insufficient sleep may lead to cognitive and emotional problems, and sleep problems are a significant factor contributing to several mental disorders [22]. Reduced access to cognitive and emotional resources due to lack of sleep may increase vulnerability to developing mental disorders [23]. Short SD and subjective sleep issues in adolescents are associated with poor MH [24]. For example, numerous studies have found that short SD significantly increases the risk of depression [25], making individuals who sleep less more susceptible to MH problems [26].

GSE has a substantial impact on human health, and individuals’ beliefs about their ability to cope with stress influence the mind-body regulatory system, affecting their physical and MH [27]. Sleep is intricately linked to adolescent psychological development [28, 29]. It plays a crucial role in MH regulation, and there is a significant overlap between poor sleep and depression [30]. Sleep affects mood regulation [31, 32], and adolescents who get sufficient sleep tend to have better MH than those who do not [33]. Adequate sleep has a positive effect on maintaining the MH of children and adolescents [34]. Sufficient SD is also a positive contributor to maintaining the MH of children and adolescents [35]. Longer SD is associated with better overall health in children [36], and it significantly impacts cognitive functioning and MH, including depression and anxiety in children [37]. Additionally, insufficient sleep is significantly associated with an increased risk of mood disorders in adolescents [38]. These studies demonstrate that SD and GSE have a positive influence on MH.

#### Other correlation studies among PA, GSE, Sleep, and MH

While research findings on the relationship between sleep deprivation and MH may vary [39], there is no consensus on the optimal level of PA to reduce negative psychiatric symptoms, considering both the frequency and type of PA [40]. The association between changes in GSE and PA behavior is minimal and statistically insignificant [41]. Although evidence of bidirectional associations between PA and sleep is limited and inconclusive, interventions addressing both PA and sleep can promote improvements in both health behaviors [42]. Notably, there is a U-shaped association between SD and the onset of depression [43]. However, there is evidence supporting the causal effect of sleep interventions in improving MH symptoms in college students [44]. Studies also advocate for a combination of sleep and PA interventions to enhance MH in populations [45].

In summary, among adolescents, there are mutually reinforcing effects among PA, SD, GSE, and MH. GSE has an indirect or mediating role in the relationship between PA and MH. Sleep quality plays a mediating role in treating chronic diseases and promoting MH [46]. It remains unclear whether SD acts as a moderator or mediator between PA and MH. Based on the relationships among SD, MH, and PA in adolescents, it is reasonable to infer that SD also plays a mediating role between PA and MH in this demographic.

### Model construction

**Fig. 1:**
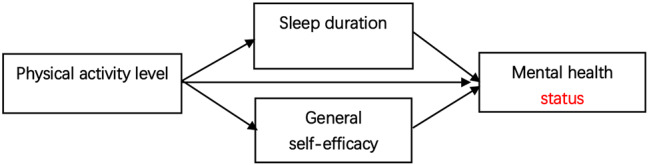
Structural diagram of physical and mental health promotion model for adolescent

In the present study, we formulated the following hypotheses:1) The PA level of adolescents has a direct promoting effect on SD, GSE, and MH; 2) SD and GSE have a direct positive effect on MH in adolescents; 3) The PA of adolescents indirectly promotes MH through the mediating effects of GSE and SD, that is, GSE and SD play a role in the association between PA levels and the psychological well-being of adolescents (see Fig. 1).

## Research methodology and investigation subjects

### Research method

#### Questionnaire survey method

##### Instruments

We employed a self-administered questionnaire concerning SD and utilized three scales as test instruments.

Scale I: The Mental Health Inventory of Middle School Students (MMHI-60) [47] employs a 5-point Likert scale with 60 items. When the total mean score is greater than or equal to 2, it indicates problematic or abnormal overall MH. This scale exhibits good reliability and validity [48]. The internal consistency reliability coefficient was Cronbach’s alpha = 0.970, and the structural validity coefficient was the Kaiser–Meyer–Olkin (KMO) score = 0.980.

Scale II: The Physical Activity Questionnaire for Children (PAQ-C) [49] was selected as it does not require detailed recall of exercise duration and intensity, reducing participant burden and recall bias. It uses a 5-point Likert scoring system [50]. The Chinese version of this questionnaire demonstrates good reliability and validity [51]. The internal consistency reliability coefficient was Cronbach’s alpha = 0.802, and the structural validity coefficient was KMO score = 0.872.

Scale III: The GSE Scale, developed by German psychologist Schwarzer, employs a 4-point Likert scale. The Chinese version exhibits good reliability and validity [52]. The internal consistency reliability coefficient was Cronbach’s alpha = 0.890, and the structural validity coefficient was KMO score = 0.923.

##### Sampling and survey

Due to COVID-19 prevention and control measures, students from different classes were not allowed to interact, so we utilized convenient random sampling. We randomly selected eight junior high schools and three senior high schools. On a rainy day when outdoor physical education classes were not feasible, the investigator randomly selected one class/grade of junior high school students and two classes/grades of senior high school students as survey respondents. The survey was conducted in the classroom using on-site distribution and collection. The questionnaires were initially reviewed before collection in November 2021. The questionnaire was administered after receiving authorization from the Academic Ethics Committee of the School of Physical Education at Guizhou Normal University and obtaining the informed consent form of parents signed by the parents of teenagers under the age of 18. The informed consent form” was reaffirmed before teenagers filled in the questionnaire.

The sample size for the social survey was calculated using the formula: N = Z^2^ × (P × (1 − P))/E^2^ [53], where N is the sample size, Z is the statistic with 95% confidence (Z = 1.96), E is 0.05, and P is 0.5. Therefore, N is approximately 384.

The expected effective response rate for the questionnaire is 80%. As adolescents aged 12–18 years are divided into two groups, junior high school students and senior high school students, the sample size for each group is greater than or equal to 482, totaling more than 964. To ensure statistical significance, the survey sample size was increased by 1.5 times. Theoretically, the sample size for junior and senior high school students should reach 723 students for each group, totaling 1446 students. Although the senior high school student sample size does not meet this requirement, the total sample number of 1491 is greater than 1446, meeting the sampling requirements.

#### Mathematical and statistical methods

Descriptive statistics regarding the responses to adolescents’ PA levels, GSE, SD, and MH status were analyzed using IBM SPSS 26.0 for Windows (IBM Corp., USA). To conduct validation analyses of structural equation models and test multiple mediating effects, we used AMOS 26.0 (IBM Corp., USA) with the Bootstrap method.

### Survey subjects

The study received approval from the Academic Ethics Committee of the School of Physical Education at Guizhou Normal University. The questionnaire survey was conducted using random and convenient sampling, with oral consent obtained from the school principals and parents of the students. We selected students from three senior high schools and eight junior high schools in Guiyang City, China, as survey participants. To ensure a one-to-one correspondence among the questionnaires for PA, GSE, and MH at the time of data analysis, the following measures were taken:

Before implementing the questionnaire, each respondent was assigned a fixed number, which they were required to fill in the upper right corner of the first page of the questionnaire and check when returning it. Whenever possible, participants were encouraged to complete all questionnaires within a single survey session. For those who needed two sessions to complete the relevant questionnaires (e.g., PAQ-C and GSE Scale as one set and SD and MMHI-60 as the other), they were instructed to use the same assigned number in the upper right corner of both questionnaires. Data entry was conducted using these assigned numbers, and the allocation table of numbers was publicly destroyed by the investigator upon completion of the questionnaire.

A total of 1491 valid questionnaires were included in the analysis. The participants consisted of 143 adolescents in grade 12, 220 in grade 11, 229 in grade 10, 268 in grade 9, 301 in grade 8, and 330 in grade 7, with 753 boys and 738 girls. The mean age of the participants was 14.94 ± 1.76 years old. Senior high school students accounted for 39.7% of the total. The number of students in each class varied among schools, with senior high school classes ranging from 25 to 40 students per class and junior high school classes having approximately 40–45 students per class. The relatively small class sizes in senior high schools contributed to the smaller sample size of senior high school students in this survey.

## Results

### Descriptive statistics of the Age, PA level, GSE, and MH of adolescents

The questionnaire survey involved a total of 8 junior high schools, with a total of 24 classes, and 3 senior high schools with 6 classes. (see Table 1).

**Table 1:** Sample distribution(sampling school, grade(class): number of students)

	The name of school	Grade(class):the number of students
1	Guizhou Normal University Affiliated Senior High School	12(1):21;12(16):18;11(7):29; 11(22):29;10(3):28;10(19):43
2	No. 6 Middle School in Guiyang	12(2):31;12(11):32;11(7):43; 11(22):44;10(3):45;10(9):44
3	No. 3 Experimental Middle School in Guiyang	12(3):20;12(11):21;11(2):41; 11(12):34;10(7):29;10(10):30
4	No. 1 Middle School of Exhibition City, Guanshanhu District in Guiyang	7(7):44;8(3):39;9(12):33
5	Guizhou Normal University Affiliated Junior High School at Gui’an District	7(2):42;8(8):36;9(1):31
6	No. 10 Middle School in Guiyang	7(4):44;8(7):37;9(2):35
7	No. 17 Middle School in Guiyang	7(5):43;8(9):38;9(2):33
8	No. 8 Middle School in Guiyang	7(1):40;8(12):39;9(6):34
9	No. 19 Middle School in Guiyang	7(2):34;8(6):38;9(5):34
10	Junior high School of East China Normal University in Guiyang	7(13):43;8(10):38;9(3):33
11	Beijing Ritan Junior high School in Guiyang	7(9):40;8(2):36;9(4):35

The sample size gradually decreases with increasing grades.

The mean PA level was 2.49 ± 0.67 (95% CI: 2.46–2.53) for adolescents, 2.65 ± 0.65 (95% CI: 2.60–2.69) for junior high school students, and 2.26 ± 0.63 (95% CI: 2.21–2.31) for senior high school students. The average SD of adolescents was 436.58 ± 71.52 min/day (95% CI: 432.95–440.21). For junior high school students, it was 465.45 ± 63.07 min/day (95% CI: 461.32–469.57), and 85.9% of junior high school students reported sleeping less than 9 h/day. For senior high school students, the average SD was 392.62 ± 60.43 min/day (95% CI: 387.74–397.50), with 89.3% of senior high school students sleeping less than 8 h/day.

The GSE scores of adolescents were 2.60 ± 6.59 (95% CI: 25.69–26.36), junior high school students had scores of 2.70 ± 6.71 (95% CI: 26.54–27.42), and senior high school students had scores of 2.46 ± 6.14 (95% CI: 24.07–25.06). The mean MH score was 1.72 ± 0.63 (95% CI: 1.69–1.76) for adolescents, 1.65 ± 0.68 (95% CI: 1.61–1.68) for junior high school students, and 1.84 ± 0.68 (95% CI: 1.79–1.90) for senior high school students. The proportion of the total MH score ≥ 2 (indicating an abnormal value) was 27.90% for adolescents, 23.3% for junior high school students, and 34.7% for senior high school students.

A pairwise correlation was observed among the PA level, SD, GSE, and total MH scores of adolescents, with all correlation coefficients being less than 0.001 (P < 0.001). Among them, there was a positive correlation between PA, SD, and GSE (P < 0.001), while all three variables were significantly negatively correlated (P < 0.001) with the total MH score. These correlations between variables provided the statistical basis and potential for testing the hypothesis model. Higher total MH scores indicated lower MH levels, yet the PA level, SD, and GSE of adolescents were positively correlated with MH (Table 2).

**Table 2:** Description statistics of SD, PA level, GSE and MH of adolescents

	Correlations of Pearson				Mean	95%CI for mean	
	SD	PAL	GSES	Total average of MH		Lower	Upper
SD	1.00				436.52	432.89	440.15
PAL	0.309**	1.00			2.49	2.46	2.53
GSES	0.141**	0.334**	1.00		2.60	2.57	2.64
MH	− 0.240**	− 0.261**	− 0.251**	1.00	1.72	1.69	1.76

*Note*: PAL = physical activity level, SD = sleep duration, GSE = general self-efficacy, MH = mental health status; ** p < 0.01

### Hypothesis model test and mediation effect analysis

**Fig. 2:**
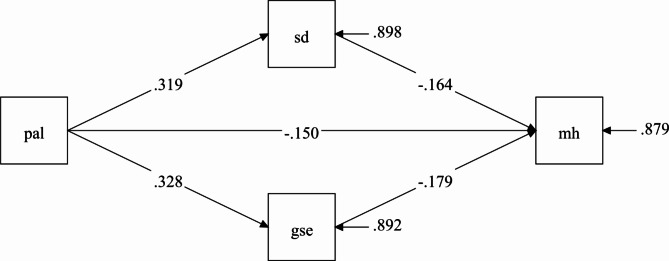
Function Path Diagram of the Model of Physical and MH Promotion for Adolescent *Note*: pal = physical activity level, sd = sleep duration, gse = general self-efficacy, mh = mental health status

Model testing revealed that the path coefficients between PA and MH, as well as between SD and GSE, were statistically significant (P < 0.001). Additionally, all fitted indicators of the constructed multiple mediation model met the corresponding statistical criteria (model fitness index: χ^2^/df = 1.295, comparative fit index = 0.999, Tucker–Lewis index = 0.997, root mean square error of approximation = 0.014, standardized root mean square residual = 0.007), indicating a good model fit (see Fig. 2).

The results suggest that the SD and GSE of adolescents play a significant mediating role between PA levels and MH status (Fig. 2). Based on the hypothesis model, we used structural equation modeling to evaluate the mediating effects of SD and GSE on PA and MH, following the mediating effect test procedure recommended by Zhonglin Wen and Baojuan Ye [54]. Our focus was on assessing the multiple mediating effects of SD and GSE on PA and MH levels in adolescents.

The bias-corrected nonparametric percentile Bootstrap test revealed that the 95% confidence intervals for the two mediating pathways did not include 0, signifying significant multiple mediating roles of SD and GSE of adolescents on their PA and MH levels. Specifically, the mediating pathway for SD was as follows: PA level ➔ SD ➔ MH (β = −0.052, P < 0.001), and the mediating pathway for GSE was as follows: PA ➔ GSE ➔ MH (β = −0.059, P < 0.001). Furthermore, PA level had a direct and significant effect on MH (β = −0.150, P < 0.001). According to the β coefficient of the two mediating pathways and the moderating effect calculation method, the moderating effect value was calculated using the following formula: [(− 0.052)+(− 0.059)]/[(− 0.052)+(− 0.059)+(− 0.150)] = (− 0.111)/[(− 0.111)+(− 0.150)] = (− 0.111)/(− 0.261) = 0.42528; this value, expressed as a percentage, is approximately 42.53%. Because mediating and direct effects are homogeneous, they are not masking effects. Therefore, in adolescents, both SD and GSE partially mediated the association between PA and MH levels, with the mediating effect accounting for 42.53% of the total effect. This suggests that almost half of the effect of the PA level of adolescents on their MH levels was mediated through the combined effects of SD and GSE. Significance tests for mediating effects indicated that GSE (53.15%) played a slightly larger role than SD (46.85%), suggesting that both SD and GSE played an incomplete mediating role in adolescents’ PA promotion of MH (see Table 3).

**Table 3:** Bootstrap analysis of the significance test for mediation effect

Indirect effect	Estimated value	Std. Error	95%CI	%
PAL∀SD∀MH	0.319 × (-0.164) = − 0.052	0.011	− 0.073 ~ − 0.031	46.85%
PAL∀GSE∀MH	− 0.198 × (-0.258) = − 0.059	0.011	− 0.081 ~ − 0.037	53.15%
Total indirect effects	− 0.111	0.014	− 0.139 ~ − 0.083	

## Discussion

The GSE levels of adolescents fall within the normal range. However, the low PA levels, significant sleep deprivation, and high rates of MH abnormalities among Chinese adolescents warrant our attention in terms of improving their physical health, addressing insufficient sleep, and enhancing their mental well-being. The mean PA level for adolescents in this study was 2.49, which is lower than that of adolescents in Beijing [50] and close to the levels of adolescents aged 12–18 years [51]. Several factors may contribute to this. First, this study was conducted during the normalization phase of COVID-19 prevention and control, which still imposed certain restrictions on people’s normal daily lives and travel. Second, approximately 40% of the respondents were senior high school students, and the average age of the participants was higher than that of the subjects in previous studies. The PA levels in children and adolescents tend to decrease with age. While the GSE Scale scores are within the normal range, it is worth noting that the GSE norm scores were slightly lower than those of European and American adolescents [52].

The overall MH score of both junior and senior high school students was lower than the norm (1.88 ± 0.57), and the overall detection rate and total average score of psychological problems among senior high school students were lower than those reported in a study from the first half of 2021 [55]. These results suggest that the overall MH of secondary school students has improved. This improvement can be attributed to several factors. First, during the normalization of COVID-19 prevention and control measures, many extracurricular training and counseling institutions were forced to close, leading to adolescents having less out-of-school study time and more discretionary time. Second, in July 2021, the Chinese government issued “*The Opinions on Further Reducing the Burden of Homework and Off-campus Training for Students in the Compulsory Education Stage”* and “*The Notice on Further Clarifying the Scope of Disciplines and Non-disciplines of Off-campus Training in the Compulsory Education Stage,”* and in September, the General Office of the Ministry of Education of the People’s Republic of China issued “*The Notice on Resolutely Investigating and Dealing with the Issue of Carrying out Off-campus Training in Disguised Violation of Regulations,”* which effectively reduce the homework burden and off-campus training burden of students in the compulsory education stage. These measures aim to provide students with more time and energy to pursue their interests and reduce the academic burden on some students.

The average sleep time and SD qualification rate of adolescents are lower than those in related studies [56, 57]. Notably, the proportion of Chinese adolescents who do not get enough sleep is higher than that of adolescents in the United States [58]. The primary reason for this serious lack of sleep among adolescents is their inclination towards using electronic devices such as mobile phones and excessive internet engagement. The results of our structural equation modeling demonstrated that the research hypotheses of this study were valid. These hypotheses included the direct impact of the PA level of adolescents on their SD, GSE, and MH. The results also confirmed the direct influence of SD and GSE on MH. Furthermore, we observed the indirect contribution of SD and GSE to the impact of PA on MH among adolescents.

Our structural model emphasizes the holistic nature of adolescent health promotion. It suggests that when planning or implementing health promotion strategies, all aspects of health issues should be considered. After understanding the interrelationships between these health elements, the content, methods, and measures of interventions should be designed for optimal effectiveness. This approach comprehensively addresses various health challenges faced by adolescents, including insufficient PA, vision health, sleep deprivation, and MH.

In particular, our study revealed that SD and GSE have multiple mediating effects on the impact of PA on MH in adolescents. This finding reaffirms the role of GSE as a mediator between PA and MH and introduces SD as an additional mediator in the relationship between PA and MH in adolescents. In contrast to a previous study [58], which suggested that sleep quality plays a mediating role in MH interventions, our study demonstrated that SD is a significant mediating factor in the process of PA influencing MH among adolescents. Notably, SD accounted for 46.85% of the mediating effect, a percentage only slightly lower than that of GSE. In the context of adolescent sleep interventions, SD is easier to measure, control, and implement than sleep quality. Therefore, our findings highlight the importance of addressing both sleep quality and SD when promoting adolescent MH. The combined mediating effects of SD and GSE accounted for 42.53% of the total effect, indicating that while we should emphasize the direct impact of adolescent PA on MH, we should not underestimate the mediating or indirect effects played by specific factors. These insights further emphasize the benefits of implementing combined interventions for PA, SD, and MH promotion among adolescents. Once again, this underscores the importance of holistic integration in the promotion of adolescent mental and physical health.

A significant limitation of this study is that the PA data relied on questionnaires, which may introduce recall bias. In future studies, it would be advisable to test the model using more objective measures, such as accelerometers.

## Conclusion

In summary, Chinese middle school students face challenges related to low levels of PA, inadequate sleep, high rates of MH abnormalities, and high GSE. Our constructed model for promoting adolescent MH through PA was shown to be effective. Both SD and GSE play crucial mediating roles in the relationship between PA and MH in adolescents. The direct impact of PA on MH is slightly greater than its indirect effects, and the regulatory effect of GSE on MH is slightly greater than that of SD. This underscores the importance of integrating PA with MH and SD interventions in adolescent health promotion work. It is also essential to focus on improving adolescents’ GSE to achieve better health promotion outcomes.

## Data Availability

The datasets generated and analyzed during the current study are not publicly available due to ethical restrictions, however, they are available from the corresponding author on reasonable request.
